# Early-Stage Pelvic Organ Prolapse and Associated Pelvic Floor Symptoms in Japanese Women Attending Routine Gynecological Screening

**DOI:** 10.7759/cureus.95921

**Published:** 2025-11-01

**Authors:** Junko Kato, Yasuhide Kitagawa, Kosei Miwa, Naoki Ito, Masanori Isobe

**Affiliations:** 1 Department of Obstetrics and Gynecology, Gifu University Graduate School of Medicine, Gifu, JPN; 2 Department of Urology, Komatsu Municipal Hospital, Komatsu, JPN; 3 Urogynecology Center, Gifu Red Cross Hospital, Gifu, JPN; 4 Department of Obstetrics and Gynecology, Chuno Kosei Hospital, Seki, JPN

**Keywords:** pelvic floor distress inventory, pelvic organ prolapse, pop-q stage, symptoms, urinary incontinence

## Abstract

Background

Pelvic organ prolapse (POP) is a common but often underrecognized condition affecting women’s quality of life worldwide. While severe or symptomatic prolapse has been the primary focus of most prior studies, there is limited information on the prevalence and symptom burden of early-stage POP in Asian populations, particularly among women who may not actively seek medical care. Establishing the presence and clinical significance of mild prolapse is essential to inform prevention, early detection, and patient education strategies.

Objective

The aim of this study was to evaluate pelvic organ support and associated pelvic floor symptoms in a large cohort of Japanese women, with particular emphasis on early-stage prolapse. We sought to clarify whether mild prolapse, often considered clinically insignificant, is in fact associated with specific and bothersome symptoms.

Methods

This single-center, cross-sectional study recruited Japanese women seeking a routine Pap smear, representing an asymptomatic or unselected general population, from July 2018 through May 2019. Women who were pregnant or had given birth in the previous six months, were using a pessary for POP, or had a history of surgery for POP or incontinence were not included. Participants completed a questionnaire covering nine symptoms based on the Japanese Pelvic Floor Distress Inventory, i.e., frequent urination, urge urinary incontinence, stress urinary incontinence (SUI), urine leakage, difficulty emptying, pain or discomfort, pelvic heaviness or dullness, vaginal bulge, and anal incontinence. Pelvic organ support was assessed by a single examiner using the Pelvic Organ Prolapse Quantification (POP-Q) system, and participants were subsequently grouped by POP-Q stage. Each vaginal compartment (anterior, posterior, apical) was assigned a stage (0-IV), with an overall stage determined on the basis of the maximum point of descensus in each participant. Relationships between compartment-specific POP stages and symptom scores were analyzed using multiple regression, with a 5% significance level.

Results

A total of 1,023 women with a mean age of 52.1 (range: 21-84) years participated in the study. The overall distribution of POP-Q stages was as follows: stage 0, 390 (38.1%); stage I, 463 (45.3%); stage II, 165 (16.1%); and stage III, 5 (0.5%). No participants had stage IV POP. Nearly half of the women (493, 48.2%) experienced symptoms of SUI, and 78 (7.6%) reported a vaginal bulge, with significant differences in symptom prevalence (chi-square test, all p < 0.05). The most common prolapse combination involved both the anterior and posterior vaginal walls, with anterior descent occurring more frequently than posterior descent. Anterior prolapse was also associated with increased SUI at ≥ stage I (p < 0.05). Vaginal bulge sensation was significantly associated with ≥ stage II prolapse in all compartments (p < 0.01). There was no association between anal incontinence and any prolapsed compartment.

Conclusion

This study represents the first large-scale evaluation in Japan to examine both POP-Q-based pelvic support and its association with pelvic floor symptoms in a screening population. Our findings provide foundational epidemiological data that bridge the gap between clinical studies of severe POP and general population surveys, offering new insight into the natural history and symptom burden of early pelvic organ prolapse.

## Introduction

Pelvic organ prolapse (POP) is a common condition that affects millions of women worldwide and significantly impairs quality of life. In Western countries, the lifetime risk of undergoing surgery for POP or urinary incontinence is estimated to be 11.1% [1]. Despite the high prevalence of POP, epidemiological data in Asia remain limited. Data on women in the general population who have not sought medical care are particularly scarce. Large-scale studies in Asia have mainly focused on symptomatic or advanced POP, rather than earlier or asymptomatic stages. For example, in a midlife cohort of women followed for 6-7 years in Singapore, 62.3% developed at least one symptom of a pelvic floor disorder [2]. In South Korea, insurance claims data were used to analyze patterns of POP diagnosis and treatment [3]. While these types of studies rely on self-reported symptoms or administrative data, standardized physical examinations employing systems such as POP Quantification (POP-Q) system [4] are utilized to a limited extent.

The relationship between POP and symptoms is complex and incompletely understood. Pelvic floor symptoms - urinary or bowel disturbances, pain or pressure - may occur even with mild prolapse or conversely be absent in some advanced cases. Indeed, many women with mild prolapse remain asymptomatic, whereas others experience noticeable discomfort even at early stages, suggesting considerable individual variation in symptom perception. In clinical practice, vaginal descent to the hymeneal ring is often regarded as “normal,” corresponding roughly to stage I and early-stage II POP according to the POP-Q system, particularly among parous or aging women [5]. However, even at these early stages, subtle descent of the anterior, posterior, or apical compartments may already influence pelvic floor symptoms. The one exception is the vaginal bulge itself: feeling or seeing a vaginal bulge is the symptom most strongly associated with higher POP stage [6]. Previous research indicates that advanced POP can cause urethral kinking, effectively masking stress urinary incontinence (SUI). Consequently, women with severe cystocele rarely exhibit SUI symptoms [7,8]. This phenomenon has been well documented in treatment-seeking patients, but little is known about how symptom patterns emerge or evolve in the general population, particularly at earlier stages of prolapse. Importantly, accumulating evidence suggests that even mild prolapse may not be entirely asymptomatic, and that early-stage conditions could already be associated with pelvic floor symptoms [9,10]. However, systematic epidemiological data addressing this question in Asian women are lacking.

In Japan, few epidemiological studies have objectively evaluated POP. Therefore, we conducted a cross-sectional study of Japanese women undergoing cervical cancer screening and reported the prevalence of POP using POP-Q system previously [11]. That prior study focused exclusively on the prevalence and risk factors of POP and did not examine symptom correlations. Building upon those prevalence data, the present investigation extends our previous work by analyzing the association between POP-Q findings and pelvic floor symptoms, providing a more comprehensive picture of pelvic floor health in this population. Identifying symptom patterns even in early-stage POP is clinically important, as it may allow timely conservative management, patient counseling, and prevention of further progression. We sought to clarify whether early-stage POP, often considered clinically insignificant, is in fact associated with specific and bothersome symptoms. Our findings provide critical insights into the early manifestations of POP in a general population.

## Materials and methods

Study design

This was a cross-sectional study conducted at the Health Checkup Center of Chuno Kosei Hospital, a regional medical institution located in the city of Seki, Gifu Prefecture, Japan. The hospital is situated in a semi-urban (regional city) area with a population of approximately 83,000.

Study population

Eligible participants were women aged ≥ 20 years who sought a Pap smear at the above facility from July 2018 through May 2019. Women who were pregnant or had given birth in the previous six months, were using a pessary for POP, or had a history of surgery for POP or incontinence were not included to avoid bias. The study included women of Japanese ethnicity who underwent cervical cancer screening. Although the facility also serves foreign residents, only Japanese women were included in the present analysis to maintain a homogeneous study population for epidemiological comparison. A formal sample size calculation was not performed; however, as approximately 1,500 women undergo screening annually at this center, the sample was considered sufficient for the intended analyses, even when accounting for those who declined to participate in the study.

Study measures

Participants were asked to fill out symptom questionnaires based on the Japanese Pelvic Floor Distress Inventory-Short Form 20 (J-PFDI-20) [12,13]. Because few participants were expected to have POP, we assessed pelvic floor symptoms through a streamlined questionnaire comprising all six items of the Urinary Distress Inventory, two items from the Pelvic Organ Prolapse Distress Inventory, and anal incontinence symptoms from the Colorectal-Anal Distress Inventory. In cases of missing responses, research assistants attempted to recontact participants to confirm answers whenever possible. Before the examination, participants were asked to empty their bladders. Following a routine gynecological assessment in the dorsal lithotomy position, each woman promptly underwent POP-Q measurement by a single examiner with experience in the POP-Q technique [4]. All points, with the exception of total vaginal length, were measured with the subject performing a maximal Valsalva effort or coughing strongly. POP was assessed at three anatomic sites: anterior wall, posterior wall, and cervix or cuff. Each compartment was evaluated separately, with the other compartments retracted using a single-bladed speculum. Participants were assigned an overall stage based on the highest stage among the three compartments, with each compartment individually staged. 

Questionnaire and symptom scoring

The participants were asked the following questions: frequent urination; urge urinary incontinence (UUI); stress urinary incontinence (SUI); small amounts of urine leakage; difficulty emptying the bladder; pain or discomfort in the lower abdomen or genital region; pelvic heaviness or dullness; sensation of a vaginal bulge; and anal incontinence. If asymptomatic, the participant’s score was 0; if symptomatic, their score ranged from 1 to 4 depending on the degree of distress. Symptom distress was scored as 1 (not at all), 2 (somewhat), 3 (moderately), and 4 (quite a bit). In this study, we did not calculate composite PFDI-20. Instead, individual item scores were used directly as outcome variables, allowing analysis of symptom-specific associations with the POP-Q stage. The exact questionnaire items used in the present study are provided in the Appendix for reference.

Ethics statement

This study was approved by the Institutional Review Board of Chuno Kosei Hospital (approval no. 70). Written informed consent was obtained from all participants prior to examinations.

Statistical analysis

Differences in the prevalence of each pelvic floor symptom were analyzed using the chi-square test. Multiple comparisons were adjusted using the Benjamini-Hochberg method. Comparison of individual inventory scores included the categorized POP-Q stages (0, I, and ≥ II) in each compartment (anterior, posterior, apical). Data were subjected to linear regression analyses. Although the symptom scores were not normally distributed, linear regression was applied because the sample size was large and the models were considered robust to minor deviations from normality. Because prolapse often occurs in two or all compartments simultaneously, we modeled independent associations of the prolapsed anatomic compartments with symptoms while controlling for prolapse in other compartments and age using multiple regression analysis. Because our examination focused on specific, predefined hypotheses regarding the associations between compartment-specific POP-Q stages and selected pelvic floor symptoms, namely anterior compartment prolapse with UUI or SUI, and posterior compartment prolapse with anal incontinence, we did not apply formal corrections for multiple comparisons. Statistical significance was defined as a p value < 0.05, with clinical plausibility taken into consideration. Data were analyzed using Statistical Product and Service Solutions (SPSS, version 29.0; IBM SPSS Statistics for Windows, Armonk, NY)

## Results

Among 1236 women examined over the study period, 1023 women aged 21 to 84 years consented to participate, yielding a participation rate of 82.8%. The demographic and clinical characteristics of the participants are shown in Table 1. There were 390 (38.1%) women with no prolapse, and no women with POP-Q stage IV prolapse. 

**Table 1:** Characteristics of the study population (N=1,023) *SD*, standard deviation, *BMI, *body mass index, *POP-Q, *Pelvic Organ Prolapse Quantification Values are presented as mean ± standard deviation (SD), median (range), or number (%). No statistical comparisons were performed for this descriptive summary.

Characteristic	Value
Age, mean ± SD (range), years	52.1 ± 11.8 (21–84)
BMI, mean ± SD, kg/m^2^	22.2 ± 3.9
Gravidity, median (range)	2 (0–7)
Parity, median (range)	2 (0–5)
Underwent hysterectomy, n	20
Baseline POP-Q stage, n (%)	
0	390 (38.1)
I	463 (45.3)
II	165 (16.1)
III	5 (0.5)
IV	0

Table 2 shows the responses and scores for individual symptoms. Among all participants, all completed the POP-Q examination and symptom questionnaire except for one woman, who did not respond to the question regarding vaginal bulge. Research assistants attempted to recontact this participant, but the missing response could not be obtained. Accordingly, this single case was excluded from the analysis of vaginal bulge symptoms, while all other analyses were conducted using complete data. The prevalence of each symptom differed significantly among questionnaire items. The most frequent issue was SUI, with 493 (48.2%) of the participants experiencing the symptom and receiving higher SUI scores compared with other conditions.

**Table 2:** Responses to questionnaires according to the Pelvic Floor Distress Inventory *SD*, standard deviation, *UUI, *urgency urinary incontinence, *SUI, *stress urinary incontinence Differences in the prevalence of each symptom were analyzed using the chi-square test with multiple comparisons (all p < 0.05). One participant who did not respond to the question on vaginal bulge was excluded from the analysis of that item.

Symptom	No (score 0), n (%)	Yes (1 or higher), n (%)	Scores (maximum 4), mean ± SD
Frequent urination	713 (69.7)	310 (30.3)	0.6 ± 1.0
UUI	784 (76.6)	239 (23.4)	0.4 ± 0.9
SUI	530 (51.8)	493 (48.2)	0.9 ± 1.0
Urinary leakage	753 (73.6)	270 (26.4)	0.5 ± 0.8
Difficulty emptying	943 (92.2)	80 (7.8)	0.1 ± 0.4
Pain or discomfort	g 907 (88.7)	116 (11.3)	0.2 ± 0.6
Heaviness or dullness	933 (91.2)	90 (8.8)	0.1 ± 0.9
Vaginal bulge	944 (92.3)	78 (7.6)	0.2 ± 1.0
Anal incontinence	853 (83.4)	170 (16.6)	0.3 ± 0.8

Table 3 presents the POP-Q stage distribution, detailing individual compartment classifications and combinations of prolapsed organs. The most common prolapse combination with ≥ stage II involved the anterior and posterior vaginal walls.

**Table 3:** Distribution of prolapsed compartments or combinations among 1,023 women *POP-Q*, Pelvic Organ Prolapse Quantification All values represent the number of participants (n).

POP-Q stage	Total	Isolated anterior	Isolated posterior	Isolated apical	Anterior and posterior	Anterior and apical	Posterior and apical	all sites
									0	390	–	–	–	–	–	–	–
I	463	162	73	54	74	52	16	32
II	165	22	19	0	62	15	5	42
III	5	0	1	0	1	2	0	1

Table 4 illustrates the relationships between symptoms across three POP-Q stage groups: stage 0 (reference group), stage I, and stages II-III. Separate comparisons for each pelvic compartment allowed for the assessment of symptom variation based on prolapse severity. To improve clinical interpretability, we additionally present the prevalence of symptoms (score ≥1) across POP-Q stages. However, the primary statistical analyses were based on symptom scores as continuous variables, as they better reflect the degree of bother. SUI inventory scores increased significantly with advancing POP-Q stages of anterior vaginal descent. Women with ≥ stage II descent in all compartments experienced a significantly stronger sensation of vaginal bulge. Because only three women had apical prolapse of ≥ stage II, these results should be interpreted with caution and are presented for reference only. No associations were found between anal incontinence and any of the POP-Q stage groups.

**Table 4:** Symptom scores and prevalence according to the POP-Q stage by vaginal compartment *POP-Q,* Pelvic Organ Prolapse Quantification,* UUI, *urgency urinary incontinence, *SUI,* stress urinary incontinence Upper rows show mean ± SD of Pelvic Floor Distress Inventory; lower rows show prevalence (score ≥1). Statistical tests were performed only on continuous scores; prevalence values are provided descriptively. *p<0.05, **p<0.01. Multiple regression models for each symptom were adjusted for age and prolapse in other compartments

Compartment	Anterior			Posterior			Apical		
POP-Q stage (n)	0 (558), ref	I (357)	II–III (108)	0 (697), ref	I (231)	II–III (95)	0 (804), ref	I (216)	II–III (3)
Symptom									
Frequent urination	0.6 ± 1.0	0.5 ± 1.0	0.6 ± 1.0	0.6 ± 0.9	0.5 ± 0.9	0.7 ± 1.0	0.6 ± 0.9	0.6 ± 1.0	1.7 ± 0.5
	30.5%	30.0%	33.3%	30.8%	26.0%	36.8%	29.5%	32.4%	100.0%
UUI	0.4 ± 0.8	0.5 ± 0.9	0.6 ± 1.1	0.4 ± 0.8	0.5 ± 0.9	0.6 ± 1.1	0.4 ± 0.9	0.5 ± 0.9	1.0 ± 1.0
	21.5%	24.1%	30.6%	22.0%	26.0%	27.4%	22.6%	25.5%	66.7%
SUI	0.7 ± 0.9	1.0 ± 1.0 **	1.3 ± 1.1**	0.8 ± 1.0	1.0 ± 1.0	1.1 ± 1.0	0.8 ± 1.0	0.9 ± 1.0	1.0 ± 1.0
	39.4%	57.1%	63.9%	44.6%	52.8%	63.2%	47.1%	51.9%	66.7%
Urinary leakage	0.4 ± 0.8	0.5 ± 0.8	0.7 ± 1.0 **	0.4 ± 0.4	0.5 ± 0.4	0.6 ± 0.5	0.4 ± 0.8	0.5 ± 0.9	0.3 ± 0.6
	23.7%	27.2%	38.0%	25.1%	28.6%	30.5%	26.0%	27.8%	33.3%
Difficulty emptying	0.1 ± 0.4	0.1 ± 0.5	0.1 ± 0.4	0.2 ± 0.6	0.2 ± 0.5	0.2 ± 0.5	0.1 ± 0.4	0.1 ± 0.4	0.3 ± 0.6
	6.5%	10.1%	7.4%	7.3%	8.7%	9.5%	8.3%	5.6%	33.3%
Pain or discomfort	0.2 ± 0.6	0.2 ± 0.6	0.1 ± 0.5	0.2 ± 0.6	0.2 ± 0.5	0.2 ± 0.6	0.2 ± 0.6	0.2 ± 0.6	0.3 ± 0.6
	10.4%	13.7%	8.3%	12.3%	8.7%	10.5%	11.6%	10.2%	33.3%
Heaviness or dullness	0.1 ± 0.5	0.1 ± 0.5	0.2 ± 0.5	0.1 ± 0.5	0.1 ± 0.4	0.2 ± 0.5	0.1 ± 0.5	0.1 ± 0.4	0.3 ± 0.6
	8.8%	8.1%	11.1%	8.6%	7.4%	13.7%	9.0%	7.9%	33.3%
Vaginal bulge	0.1 ± 0.4	0.1 ± 0.4	0.3 ± 0.8 *	0.1 ± 0.4	0.1 ± 0.5	0.3 ± 0.7 *	0.1 ± 0.5	0.1 ± 0.4	2.3 ± 2.1 **
	6.3%	8.1%	13.0%	6.6%	7.8%	14.7%	7.7%	6.5%	66.7%
Anal incontinence	0.3 ± 0.7	0.3 ± 0.7	0.2 ± 0.6	0.3 ± 0.7	0.2 ± 0.5	0.3 ± 0.7	0.3 ± 0.7	0.2 ± 0.7	0.7 ± 1.2
	16.3%	17.6%	14.8%	17.5%	12.6%	20.0%	17.5%	13.0%	33.3%

## Discussion

This study is the first large-scale documentation of POP-Q stage distribution and symptoms assessed through physical examination in Japan. In our cohort, 170 (16.6%) of women seeking a routine Pap smear were found to have POP ≥ stage II, which is clinically defined by the International Continence Society as POP reaching or extending beyond the hymen [14]. Previous studies of Asian patients have reported low prevalence rates of POP. These include a symptomatic POP prevalence of 9.6%, based on self-reported bulge symptoms in a Chinese survey [15], and 71 cases of POP per 100,000 hospitalized patients in a study based on Korean insurance claims data [3]. Unlike prior research focusing on symptomatic or surgically treated patients, we assessed a general population of women undergoing a routine gynecological checkup. Although many women with stage II POP are asymptomatic and may not require intervention, our finding that a substantial proportion of this population met the clinical definition of POP underscores the possibility that early-stage prolapse may go unrecognized in routine clinical settings. As healthcare providers, we should acknowledge the presence of women with unrecognized POP in the general population and emphasize the need for greater awareness and education regarding early pelvic floor changes.

Existing data suggest that ethnicity may influence the prevalence of POP. In a cross-sectional analysis of the Women’s Health Initiative Hormone Replacement Therapy trial, Hispanic women had the highest risk of uterine prolapse, Asian women had the highest risk of cystocele and rectocele, and African American women had the lowest risk of all types of prolapse [16]. In our cohort, anterior and posterior wall prolapse was the most prevalent form of POP ≥ stage II. We also found that anterior descent occurred more frequently than posterior descent, aligning with previous Asian studies that identified anterior compartment prolapse as the most prevalent subtype [17], consistent with data from a nationwide population-based survey in China [15]. This trend may reflect anatomical differences and lifestyle-related factors specific to Asian women. Our distribution of pelvic organ support demonstrated that known ethnic differences in POP may already be observed at the early stages of prolapse.

As expected, 853 (approximately 80%) of our gynecological checkup attendees were classified as POP-Q stage 0 or I, indicating normal vaginal support. Nevertheless, nearly half of the participants reported symptoms of SUI. Building on this observation, we examined the relationship between early POP and urinary symptoms based on a predefined hypothesis. While frequent urination and UUI showed no association with prolapse staging, we found that even a slight descent of the anterior vaginal wall increased SUI inventory responses and urinary leakage. Despite previous reports of an inverse relationship between SUI frequency and POP stage, the observed positive association between POP and SUI may reflect the small number of women with severe POP in our study population. Furthermore, when the six urinary items were summarized as a composite UDI-6 subscore, the mean total score was significantly higher among women with anterior vaginal descent at stage I (p < 0.05) and ≥ stage II (p < 0.01) compared with those at stage 0. These findings reinforce that early anterior prolapse is associated with greater urinary symptom burden. Recent recommendations from the French High Authority of Health (HAS) on POP management further emphasize the importance of pelvic examination in women presenting with POP-related symptoms, in order to exclude other possible conditions [18]. By quantifying these relationships in an ostensibly healthy group, the study provides novel evidence that early-stage prolapse can contribute to daily symptoms for women.

Some of the women in our study reported POP-specific symptoms, with 78 (7.6%) overall reporting the sensation of a vaginal bulge. Similarly, a previous study found a vaginal bulge prevalence of 8.7% in a general population [9]. In this study, women with a history of treatment for POP were excluded. Our findings revealed that a certain proportion of women still experience these symptoms. Although these symptoms may be difficult to discuss openly in a clinical setting due to embarrassment, their presence indicates that unreported cases exist in the general population. This highlights the importance of recognizing that even women who do not seek medical care may suffer from pelvic floor symptoms. The inventory response of vaginal bulging increased significantly in ≥ stage II prolapse across all compartments. While stage II prolapse is often not an indication for surgery, its progression remains uncertain, emphasizing the importance of educating women about preventive care, such as physical or behavioral therapy. Conservative interventions, especially pelvic floor training, have been shown to improve symptoms even in women with mild POP [19]. Increasing awareness and access to information about POP will empower women to seek help and consult an appropriate healthcare provider when symptoms arise.

The strength of the present study lies in its detailed assessment of pelvic organ support, utilizing POP-Q-based diagnosis by a single examiner. This approach minimized interobserver variability and ensured consistent staging across participants. The examiner’s diagnostic validity and reproducibility of POP-Q measurements had been evaluated in a previous pilot study [20], supporting the reliability of the assessment method used in the present investigation. Furthermore, to our knowledge, this is the first large-scale study in a Japanese screening population that combined objective assessment with systematic symptom evaluation, thereby demonstrating that clinically relevant associations can already be observed at the earliest stages of prolapse. These results provide new epidemiological data on mild POP in an Asian population, helping to fill the gap between clinical studies of severe POP and population surveys on symptomatic prolapse. However, we also acknowledge several limitations of this study. First, there is potential selection bias, as the study population comprised women undergoing routine gynecological examination, which may not reflect the general population. Indeed, the rate of women undergoing Pap smear screening in Japan is relatively low (43.6%) [21], further limiting representativeness. Additionally, concerns about generalizability arise from the fact that this was a single-center study conducted in Gifu, Japan. Women in rural areas or other countries may exhibit different POP profiles. Furthermore, only five women in our cohort had stage III or greater POP, limiting the ability to conduct statistical comparisons or derive robust conclusions regarding advanced prolapse. The masking of SUI and the symptoms caused by bladder wall stretching in severe cystocele could not be adequately evaluated.

## Conclusions

This study represents the first large-scale assessment of the relationship between POP-Q-based pelvic support and symptoms in a Japanese screening population. We demonstrate that even POP of stage I can be linked with specific symptoms, challenging the assumption that mild prolapse is clinically irrelevant. In clinical practice, recognition of symptomatic early-stage POP underscores the importance of early pelvic floor care and awareness-based education. At the population level, given the aging demographic and low awareness of pelvic floor disorders in Japan, strategies for early detection and knowledge dissemination are essential to improve women’s health outcomes. These findings may also serve as a foundation for future international comparisons and longitudinal studies aimed at refining prevention and management strategies for pelvic floor disorders.

## Appendices

Instructions: These questions will ask you if you have certain bowel, bladder, or pelvic symptoms and, if you do, how much they bother you. Answer these by putting an ✔︎ in the appropriate box or boxes. While answering these questions, please consider your symptoms over the last 3 months.

All items use the following format with a response scale from 0 to 4.

Do you _______________________?　　

☐ No; ☐ Yes　

If yes, how much does it bother you?　

              ☐ 1    ☐ 2    ☐ 3    ☐ 4　

Not at all   Somewhat  Moderately  Quite a bit
　

1. Usually experience frequent urination?

2. Usually experience urine leakage associated with a feeling of urgency, that is, a strong sensation of needing to go the bathroom?

3. Usually experience urine leakage related to coughing, sneezing, or laughing?

4. Usually experience small amounts of urine leakage (that is, drops)?

5. Usually experience difficulty emptying your bladder?

6. Usually experience pain or discomfort in the lower abdomen or genital region?

7. Usually experience heaviness or dullness in the pelvic area?

8. Usually have a bulge or something falling out that you can see or feel in your vaginal area?

9. Usually lose stool or gas beyond your control?
